# EzSkiROS: enhancing robot skill composition with embedded DSL for early error detection

**DOI:** 10.3389/frobt.2024.1363443

**Published:** 2025-01-03

**Authors:** Momina Rizwan, Christoph Reichenbach, Ricardo Caldas, Matthias Mayr, Volker Krueger

**Affiliations:** ^1^ Department of Computer Science, Faculty of Engineering (LTH), Lund University, Lund, Sweden; ^2^ Department of Computer Science and Engineering, Chalmers University of Technology, Gothenburg, Sweden

**Keywords:** embedded domain-specific languages, robot skills, skill-based control platforms, behavior trees, domain-specific language design patterns

## Abstract

When developing general-purpose robot software components, we often lack complete knowledge of the specific contexts in which they will be executed. This limits our ability to make predictions, including our ability to detect program bugs statically. Since running a robot is an expensive task, finding errors at runtime can prolong the debugging loop or even cause safety hazards. This paper proposes an approach to help developers catch these errors as soon as we have some context (typically at pre-launch time) with minimal additional efforts. We use embedded domain-specific language (DSL) techniques to enforce early checks. We describe design patterns suitable for robot programming and show how to use these design patterns for DSL embedding in Python, using two case studies on an open-source robot skill platform SkiROS2, designed for the composition of robot skills. These two case studies help us understand how to use DSL embedding on two abstraction levels: the high-level skill description that focuses on what the robot can do and under what circumstances and the lower-level decision-making and execution flow of tasks. Using our DSL EzSkiROS, we show how our design patterns enable robotics software platforms to detect bugs in the high-level contracts between the robot’s capabilities and the robot’s understanding of the world. We also apply the same techniques to detect bugs in the lower-level implementation code, such as writing behavior trees (BTs), to control the robot’s behavior based on its capabilities. We perform consistency checks during the code deployment phase, significantly earlier than the typical runtime checks. This enhances the overall safety by identifying potential issues with the skill execution before they can impact robot behavior. An initial study with SkiROS2 developers shows that our DSL-based approach is useful for finding bugs early and thus improving the maintainability of the code.

## 1 Introduction

The design and implementation of robotic systems to perform socio-technical missions have never been more relevant or challenging. To ensure that robot developers can meet market demands with confidence in the correctness of their systems, a range of development tools and techniques is required. Specifically, robot development tools should provide expressive programming languages and frameworks that allow human developers to describe correct robot behavior (Brugali et al., 2007). One such robot development platform is *SkiROS2* ^1^, a skill-based robot control platform with knowledge integration. *SkiROS2* (Mayr et al., 2023b) allows developers to define modular skills for autonomous mission execution.

These skills, ranging from “pick” to “drive,” are modularly defined with pre- and post-conditions. In SkiROS2, the assessment and validation of these conditions rely on the robot’s knowledge, systematically organized into an ontology. These *ontologies* are a rich, interlinked representation of concepts and relationships within a specific domain. They serve as a foundation for verifying that all necessary conditions for skill execution are satisfied. For instance, in an automated assembly line or robotic healthcare surgery, the ontology would encompass all relevant entities and their relationships, providing a comprehensive context for skill execution.

**FIGURE 1:**
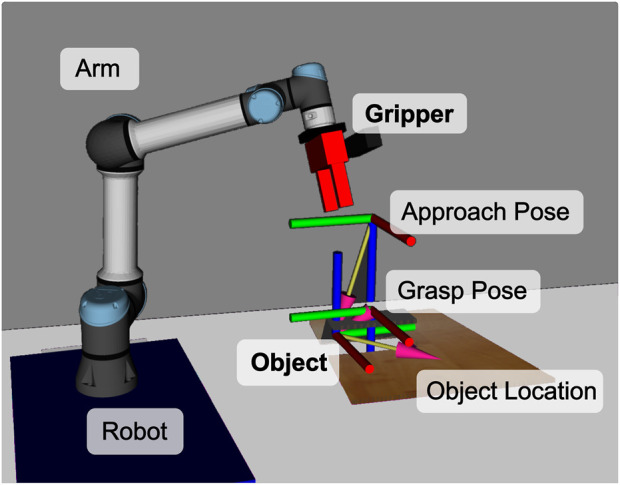
Robot using a pick skill with a visualization of the necessary parameters. To run this skill, we only need the Gripper and the Object parameters. SkiROS2 can deduce all other necessary parameters through a set of rules in the skill description shown in listings 4 and 6.

Consider a scenario where the robot has to pick an Object with its Gripper as shown in Figure 1. The pre-conditions of a “pick” skill might include ontology-based relationships such as “*the gripper is part of the robot arm*.” This relationship assists in deducing additional parameters such as “*which arm to move*” by employing subtle semantic differences of entities and their relationships in the ontology. For example, if we say that the gripper is part of the arm, then we know which arm to move if we want to pick an object with the gripper. The distinction between relationships like “*is part of*” and “*is holding*” is critical in ensuring the correct application of parameters and actions during skill execution.

The developer must be careful when declaring such relationships as bugs introduced at this stage can lead to silent errors, disrupting the skill’s behavior and potentially leading to incorrect or inefficient task execution. The reason is that some of these errors in the skill description are logical errors that would not manifest themselves as explicit runtime errors. Certain errors may only become evident when a particular skill is executed, which could be weeks later when demonstrating the robot under specific circumstances that are not immediately predictable. This delay in detection makes troubleshooting and rectifying these errors more challenging. Therefore, properly defining relationships and conditions within the ontology and skill descriptions is crucial to ensure the technical correctness and operational reliability of robotic skills in real-world applications.

In SkiROS2, each high-level skill description acts as a behavioral contract, setting parameters and conditions that the corresponding implementations must satisfy. These descriptions guide the development of concrete skill implementations. Many implementations use extended behavior trees (BTs) that reuse other existing skills, relying on their pre-conditions and post-conditions for a structured execution. Extended BTs in SkiROS2 merge task-level planning and execution, allowing for modularity and reactivity (Rovida et al., 2017b). The reactivity stems inherently from BTs in the way with which tasks are organized, which defines their priority order, with more important tasks interrupting less important tasks (Iovino et al., 2022). However, constructing consistent and correct BTs is crucial as inconsistencies can lead to unexpected failures and outcomes.

To avoid such errors, we propose using a domain-specific language (DSL) to allow the code to be analyzed for potential errors before deploying it on the actual robot. Our proposed approach ensures that the high-level abstract skill descriptions align with the lower-level BTs, providing a comprehensive framework for skill execution. DSLs offer specific constructs for defining and connecting nodes, conditions, and actions, enforcing correct patterns and practices, thus reducing the likelihood of logical or structural errors. The benefits of using DSLs to aid in debugging, visualization, and static checking are well recognized, making them a valuable tool in robot software development. DSLs have been used for mission specification (Dragule et al., 2021) and modeling of robot knowledge (Ceh et al., 2011). Nordmann et al. (2016) collected and categorized over 100 such DSLs for robotics in their *Robotics DSL Zoo* ^2^.

We aim to support robot developers, particularly those who write control logic in Python, in catching bugs early by embedding DSLs directly in Python. We support our case through the following ways:

• Four design patterns for *embedding DSLs in general-purpose programing languages* that address common challenges in robotics, with details on how to implement these patterns in Python.
• A case study of a robotics software SkiROS2, in which we introduce our DSL EzSkiROS for early detection of type errors and other bugs, highlighting its effectiveness in identifying errors in both high-level skill descriptions and lower-level implementation details.
• A demonstration of how EzSkiROS detects various types of bugs in robot capabilities, world model contracts, and behavior trees, showcasing the DSL’s comprehensive coverage and versatility in early detection of bugs early.

Lastly, we discuss the advancements and distinctions of our approach compared to the initial insights presented in the paper Rizwan et al. (2023), providing an overview of the evolution and impact of our design patterns.

## 2 Related work

Several studies have explored the use of model-driven approaches for programming robots, focusing on the development of DSLs to enhance the reliability of robotic systems. Buch et al. (2014) described an internal DSL technique written in C++, which incorporates structuring of complex actions, where actions are modeled through sets of parameters, and each action contains a pre-condition specifying the state of relevant parts. This structure implies the use of pre- and post-conditions in sequencing robotic skills. Unlike our DSL, their DSL uses a model-driven approach, which instantiates the textual representation of the assembly sequence, which is interpreted to execute the assembling behavior. However, it is unclear if they use early checking techniques to prevent erroneous sequences. Although it discusses error handling and the probabilistic approach to tackle uncertainties, specific methods like early checking techniques are not clearly outlined.

Kunze et al. (2011) proposed the Semantic Robot Description Language (SRDL), a model-based approach that utilizes the Web Ontology Language (OWL) notation to match robot descriptions and actions through the static analysis of robot capability dependencies. SRDL models the knowledge about robots, capabilities, and actions, contributing to the understanding and specification of robotic behaviors. However, the extent to which SRDL supports early dynamic checking in general-purpose languages remains unclear, highlighting the need for further exploration in this area.

Coste-Maniere and Turro (1997) proposed MAESTRO, an external DSL for specifying the reactive behavior and checking in the robotics domain. MAESTRO focuses on complex and hierarchical missions, accommodating concurrency and portability requirements. It allows the specification of user-defined typed events and conditions, offering type-checking of user-defined types and stop condition checks to ensure the correctness and safety of specified behaviors.

Behavior trees have emerged as an effective method to model and execute autonomous robotic behaviors, particularly in dynamic environments. Unlike the traditional finite-state machines (FSMs), BTs represent action selection decisions in a hierarchical tree structure enhancing the flexibility in planning and replanning robotic behavior. Dortmans and Punter (2022) highlighted that BTs offer a more maintainable approach to decision-making than FSMs, which is crucial in the rapidly evolving field of robotics. Originally developed for the video game industry, BTs have been widely adopted in robotics due to their modularity and scalability. Iovino et al. (2022) presented a detailed survey of BTs in robotics and AI, discussing their application, evolution, and benefits. BTs are composed of various types of nodes, including control nodes (e.g., sequences and selectors), leaf nodes (e.g., tasks and conditions), and decorator nodes (modifying the behavior or output of other nodes), organized in a tree structure from a root node and branching out.

Integration of BTs with robotic systems often involves the use of DSLs and frameworks such as the robot operating system (ROS). Ghzouli et al. (2023) emphasized the growing use of BTs in open-source robotic applications supported by ROS, indicating their practicality in the real-world applications. However, verifying the safety and correctness of BTs remains a challenge.

Henn et al. (2022) used SMTs to check safety properties specified in the linear constraint Horn clause notation over behavior tree specifications. Moreover, Tadiello and Troubitsyna (2022) used Event-B for the formal specification and verification of BT instances, ensuring the maintenance of invariant properties.

From a static semantics perspective, BhTSL is an example where the compiler checks the source text for non-declared variables and variable redeclaration (Oliveira et al., 2020). Despite the advancements in BT DSLs, there is a lack of DSLs performing static checks as rigorously as desired. According to the survey paper (Ghzouli et al., 2020), the most used behavior tree DSLs, such as BehaviorTree.CPP^3^, py_trees^4^, and the behavior tree from Unreal Engine^5^, primarily focus on runtime type safety and flexibility. For instance, in the MOOD2Be’s^6^ project from Horizon 2020, the BehaviorTree.CPP tool offers a C++ implementation of BTs with type safety (Faconti, 2019), but the type-checking capability is largely left to the developer and is subject to runtime checks. This indicates a gap in the domain of DSLs for BTs in ensuring correct execution behavior and preventing inconsistencies in the implementation between the skills or actions in a BT before runtime.

In conclusion, although there have been significant advancements in DSLs for robotics and BTs, there is a continuous need for the development of languages and tools that allow for both static and early dynamic checks to ensure the safety, reliability, and efficiency of robotic systems. Future research should focus on enhancing the capabilities of DSLs to perform comprehensive checks and verification, both at design time and runtime, to address the increasing complexity and demands of modern robotic applications.

## 3 Embedding robotics DSLs in Python

Domain-specific languages can help developers by simplifying the notation, improving performance, or through early error detection. However, development and maintenance of DSLs requires effort. For *external DSLs* (e.g., MAESTRO and SRDL), much of this effort comes from building a language frontend. *Internal* or *embedded DSLs* [as shown in Buch et al. (2014)] avoid this overhead and instead re-use an existing “host” language, possibly adjusting the language’s behavior to accommodate the needs of the problem domain.

We consider Python as one of the three main languages supported by the popular robotics platform ROS (Quigley et al., 2009). The other two languages, C++ and LISP, also support internal DSLs, but with different trade-offs.

### 3.1 Python language features for DSLs

Although Python’s syntax is fixed, it offers several language constructs that DSL designers can repurpose to reflect their domain, such as freely overloadable infix operators (excluding type-restricted Boolean operators), type annotations (since Python 3.0), and runtime reflection.

Listing 1 illustrates how the Python code can use these three techniques. Here, class MagicDict inherits from Python’s built-in dict class (representing mutable finite maps or associative arrays) and defines two Python functions. An instance of this MagicDict class behaves almost entirely like a regular dict, meaning that we can, e.g., read from and write to its elements (Listing 2, lines 2–5).

The first Python function we define in MagicDict is __getattribute__ (Listing 1, lines 4–11), which is a special operation that Python uses to resolve the names of attributes (meaning fields and methods) in an object. If m is a MagicDict, then whenever we read from a field of m (e.g., when we evaluate m.f), Python calls m.__getattribute__(‘f’), which defaults to an internal mechanism in Python that reads out the value of the field of that name or raises an exception. Our implementation overrides this behavior and extends it: whenever we are reading or calling an attribute that is not defined or inherited in the MagicDict class, our code instead interprets the attribute name as a key of the underlying dictionary (lines 10–11). We see the effect of this behavior in Listing 2, lines 5 and 6: our MagicDict allows us to use m.foo as an alternative to m[’foo’] to look up the key ’foo’ in the MagicDict m.

Listing 1 An example of DSL-friendly Python features: Lines 4–11 show runtime reflection, and line 14 shows a custom infix operator definition and a type annotation.

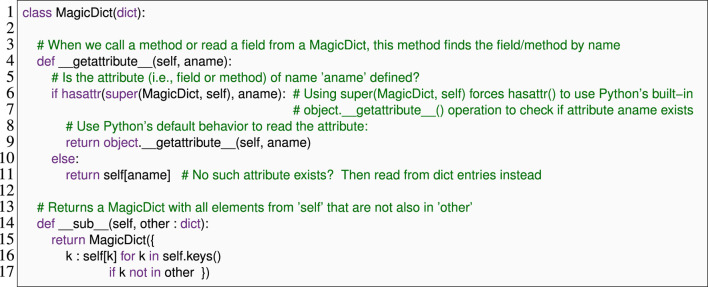

Listing 2 Interactive use of the MagicDict class from Listing 1. Lines 1–4 demonstrate standard dict features.

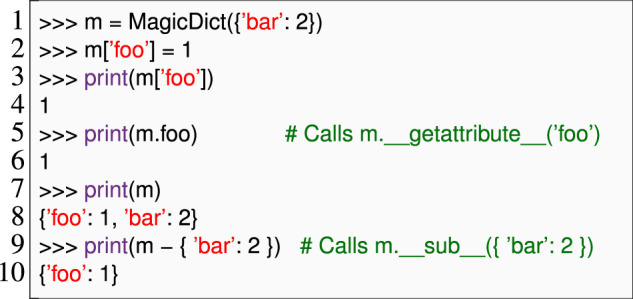

Class MagicDict overloads the infix subtraction operator in line 13 and defines an operation that allows “subtracting” a dict from a MagicDict. Our implementation is quite simplistic: if m1 is a MagicDict and m2 is a dict or an object that behaves similarly, then m1 - m2 returns a copy of m1, but without any keys that are also present in m2, as shown in Listing 2; lines 9–10.

Line 13 also illustrates Python’s type annotations, annotating the parameter other with type dict. By default, such annotations have no runtime effect, but DSL designers can access and repurpose them to collect DSL-specific information without interference from Python. With Python 3.5 (with extensions in 3.9), these annotations also allow type parameters (e.g., x: list [int]).

Python also permits the dynamic construction of classes (and metaclasses), which we have found particularly valuable for the robotics domain; since the system configuration and world model used in robotics are often specified outside of Python (e.g., in configuration files or ontologies) but are critical to program logic, we can map them to suitable type hierarchies at robot pre-launch time (just after build time).

### 3.2 Robotics DSL design patterns

In the following section, we list our DSL design patterns. A brief summary that highlights each pattern's purpose and key implementation concepts can be found in Table 1.

**TABLE 1:** Patterns summary.

Pattern	Purpose	Implementation
Domain language mapping	Make domain notation visible in host language and reduce notational overhead	See the “Piggyback” DSL implementation pattern documented by Spinellis (2001)
Staged verification	Detect type and configuration errors in a critical piece of code early, such as during robot pre-launch time, with no or minimal extra effort for developers	Execute all critical pieces of code early, while redefining the semantics of the predetermined set of operations (e.g., ontology relations from our previous example) to immediately return or to only perform checking
Symbolic tracing	Detect bugs in a critical piece of code early, if that code depends on parameters or operation return values, with minimal extra effort for developers	Execute the critical code while passing symbolic values as parameters and/or returning symbolic values from operations of relevance
		Collect any constraints imposed by operations on the symbolic values
		After executing the critical code, verify the constraints against the problem domain
Source provenance tracking	Make early dynamic error reports more actionable by reporting relevant source locations	Dynamic stack inspection

#### 3.2.1 Domain language mapping

Domain language mapping identifies language concepts in the host language that correspond to the domain language in some sense and then uses the techniques described in Spinellis (2001) to implement them. This mapping can be manual or the result of reflection.

As an example, the Web Ontology Language (OWL) allows us to express the relationships and attributes of the objects in the world, the robot hardware, and the robot’s available capabilities (skills and primitives). Existing libraries like *owlready2* (Lamy, 2017) already expose these specifications as Python objects, so if the ontology contains a class pkg:Robot, we can create a new “robot” object by writing

r = pkg.Robot ("MyRobotName")

and iterate over all known robots by writing

for robot in pkg.Robot.instances (): …

Although Moghadam et al. (2013) expressed concerns about “syntactic noise” for DSL embedding in earlier versions of Python, when compared to external DSLs, we found such noise to be modest in modern Python and instead emphasize the advantages of embedding in a language that is already integrated into the ROS environment and developers are familiar with.

##### 3.2.1.1 Maintenance and integration considerations

When domain knowledge is available in the machine-readable form, much or all of the mapping process may be automatable. For example, the *owlready2* library creates these classes at runtime based on the contents of the ontology specification files. Thus, changes in the ontology are immediately reflected in Python; if we rename pkg:Robot in the ontology, our earlier code example will trigger an error when it encounters pkg.Robot in the Python source code.

Another strategy for automating the mapping process is to generate the code in the host language. In our example, this code would take the form of Python modules, such as pkg.py, which contain classes and methods to reflect the mapping (e.g., a class Robot). This strategy mirrors the DSL implementation strategies for host languages that lack advanced reflection facilities, such as C (Levine et al., 1992).

Code generation has two potential disadvantages over reflection. First, code generation persists a *snapshot* of the domain language mapping. The build and development process must thus ensure that this snapshot is kept fresh and prevents developers from accidentally modifying the generated code. Second, code generation requires the domain language mapping to take place *before* build time. When the domain knowledge is only available at pre-launch time, the generated code will necessarily be stale, which may render this implementation strategy useless.

In our discussions with practitioners, we did however observe a key advantage that code generation offers. Since the mapping becomes visible as the Python source code, it is also available to language servers and integrated development environments and may help developers find bugs in their code even earlier.

#### 3.2.2 Staged verification

Staged Verification verifies certain kind of properties in a critical piece of code at an early stage before execution. The term “staged” refers to performing these verifications in a controlled manner at a specific early point in the process. This approach prevents runtime failures, simplifies debugging, and enables safe validation in systems that integrate complex elements. In tools like SkiROS2, combining Python code, ontologies, and configuration files at runtime introduces points of failure. To detect such failures early, we propose the following second pattern:

Listing 3 Constructing the behavior tree of a drive skill in SkiROS2:It is a sequential execution of a compound skill (a skill with its ownBT of smaller, executable skills) “Navigate” and a primitive skill (an atomic skill that cannot be broken down into smaller parts) to update the world model “WmSetRelation.”

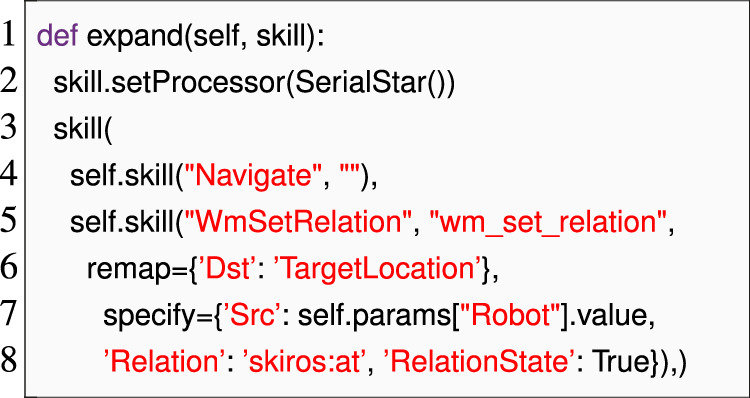

The *conditions* for this pattern are as follows:

• We can collect all critical pieces of code at a suitably early point during execution.
• The critical code does not depend on return values of operations that we cannot predict at the pre-launch time.

In Python, configuration and type errors only trigger software faults once we run the code that depends on faulty data. In robotics, we might find such code in operations that (a) run comparatively late (e.g., several minutes after the start of the robot) and (b) are difficult to unit-test (e.g., due to their coupling to specific ROS functionality and/or robotics hardware). For robotics developers, both challenges increase the cost of verification and validation (Reichenbach, 2021). A fault might trigger only after a lengthy robot program and require substantial manual effort to reproduce. For example, a software module for controlling an arm might take a configuration parameter that describes the target arm pose. If the arm controller is triggered late (e.g., because the arm is part of a mobile platform that must first reach its goal position), any typos in the arm pose will also trigger the fault late. If the pose description comes from a configuration file or ontology, traditional static checkers will also be ineffective. We can only check for such bugs after we have loaded all relevant configurations.

Through careful software design, developers can work around this problem, e.g., by checking that code and configuration are well-formed as soon as possible, before they run the control logic. If the critical code itself is free of external side effects, the check can be as simple as running the critical code twice. For example, SkiROS2 composes *BTs* (Colledanchise and Ögren, 2018) within such critical Python code (Listing 3); composing (as opposed to running) these objects has no side effects, so we can safely construct them early to detect simple errors (e.g., typos in parameter names). This is a typical example that eludes static checking but is amenable to early dynamic checking. Line 7 depends on self.params [“Robot”].value, which is a configuration parameter that we cannot access until the robot is ready to launch.

Not all of the robotics code is similarly declarative. Consider the following example, in a hypothetical robotics framework in which all operations are subclasses of RobotOp and must provide a method run () that takes no extra parameters.


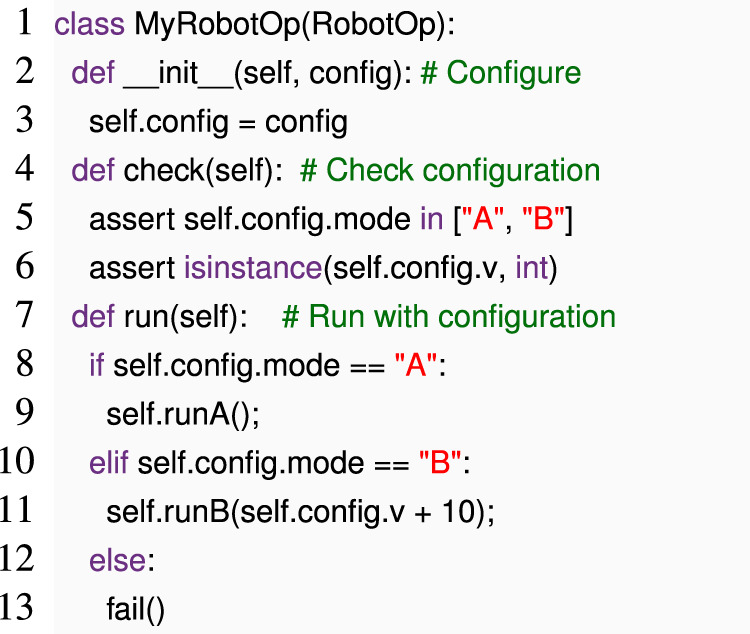


Here, developers introduced a separate method check () that can perform an early check during robot initialization or pre-launch. However, check () and run () both have to be maintained to make the same assumptions.

The early dynamic checking pattern instead uses internal DSL techniques to enable developers to use the same code in two different ways: (a) for checking and (b) for logic.

In our example, calling run () “normally” captures case (b). For case (a), we can also call run (), but instead of passing an instance of MyRobotOp, we pass a *mock* instance of the same class, in which operations like runA () immediately return.


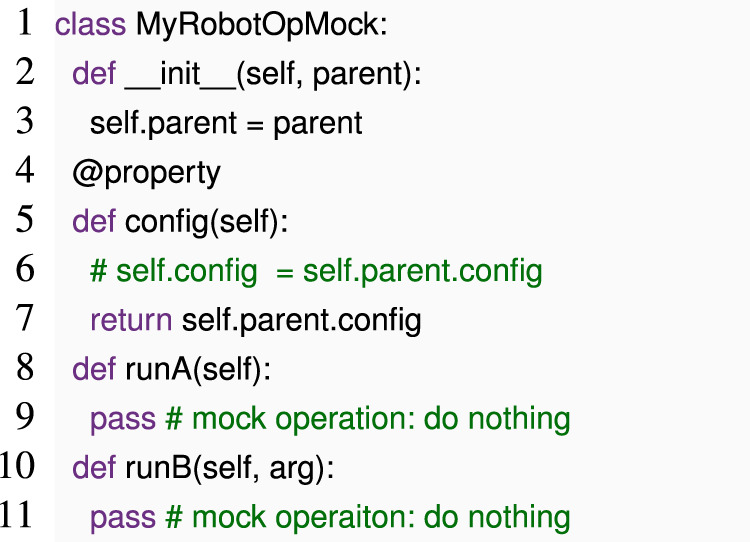


If we execute MyRobotOpMock.run () with the same configuration as MyRobotOp, run () will execute almost as for MyRobotOp but immediately return from any call to runA or runB. If the configuration is invalid, for example, if config.mode == “C” or config.v == false, running MyRobotOpMock.run () will trigger the error early.

Since Python can reflect on a class or an object to identify all fields and methods, we can construct classes like MyRobotOpMock at runtime; instead of writing them by hand, we can implement a general-purpose mock class generator that constructs methods like runA and accessors like config automatically. If the configuration objects trigger side effects, we can apply the same technique to them.

However, the above implementation strategy is only effective if we know that the critical code will only call methods on self and other Python objects that we know about ahead of time. We can relax this requirement by controlling how Python resolves nonlocal names:^7^

FunctionType(MyRobotOp.run.__code__, globals() | {‘print’: g})(obj)

This code will execute obj.run () via the equivalent MyRobotOp.run (obj) but replace all calls to print by calls to some function g. The same technique can use a custom map-like object to detect at runtime which operations the body of the method wants to call and handle them suitably.

However, the more general-purpose we want the critical code to be, the more challenging it becomes to apply this pattern. For instance, if the critical code can get stuck in an infinite loop, so may the check; if this is a concern, the check runner may need to use a heuristic timeout mechanism. A more significant limitation is that we may not, in general, know what our mocked operations like runA () should return, if anything. If the critical code depends on a return value (e.g., if it reads ROS messages), the mocked code must be able to provide suitable answers. The same limitation arises when the critical code is in a method that takes parameters. If we know the type of the parameter or return value, e.g., through a type annotation, we can exploit this information to repeatedly check (i.e., *fuzz-test*) the critical code with different values; however, without further cooperation from developers, this method can quickly become computationally prohibitive.

If we know that the code in question has a simple control flow, we may be able to apply the next pattern, symbolic tracing.

#### 3.2.3 Symbolic tracing

Here, a *symbolic* value is a special kind of mock value that we use to record information (King, 1976).

The *conditions* for this pattern are as follows:

• We can access and execute the critical code.
• We have access to sufficient information (via type annotations and properties) to simulate parameter values and operation return values *symbolically* (see below).
• The number of control flow paths through the critical code is small (see below).

Consider the following RobotOp subclass:


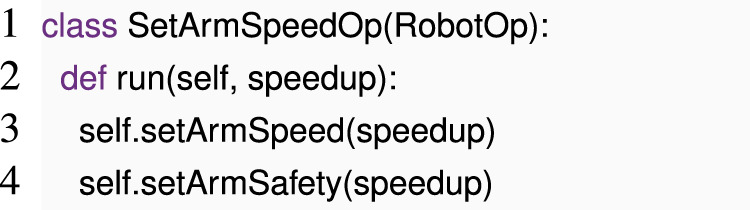


This class only calls two operations, but its run operation depends on a parameter speedup about which we know nothing a priori—thus, we cannot directly apply the early dynamic checking pattern.

In cases where we lack prior knowledge about an operation, it may still be possible to obtain useful insights about it. For example, if we are aware that setArmSpeed accepts only numeric parameters and setArmSafety only accepts Boolean parameters, we can flag this code as having a type error. To avoid blindly testing various parameters, we can pass a symbolic parameter to the run function and employ a modified version of the mock-execution strategy used in early dynamic checking. The mock objects can be adapted as follows:


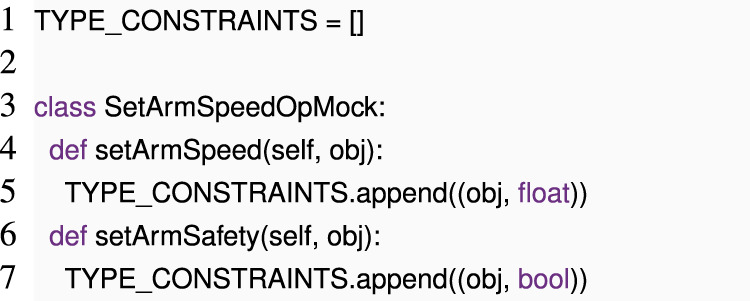


We can now 1) create a fresh object obj and an SetArmSpeedOpMock instance that we call mock, 2) call SetArmSpeedOp.run (mock, obj), and 3) read out all constraints that we collected during this call from TYPE_CONSTRAINTS and check them for consistency, which makes it easy to spot the bug. If the constraints come from accesses to obj (e.g., method calls like obj.__add__(1) that result from code like obj + 1), obj itself can collect the resultant constraints.

Depending on the problem domain, constraint solving can be arbitrarily complex, from simple type equality checks to automated satisfiability checking (Balldin and Reichenbach, 2020). It can involve dependencies across different pieces of the critical code (e.g., to check if all components agree on the types of messages sent across ROS channels or to ensure that every message that is sent has at least one reader). However, this approach requires information about specific operations like setArmSpeed and setArmSafety, which can be provided to Python in a variety of ways, e.g., via type annotations.

As an example, consider an operation that picks up a coffee from the table with a gripper, where we annotate all parameters to run with the Web Ontology Language (OWL) ontology types.


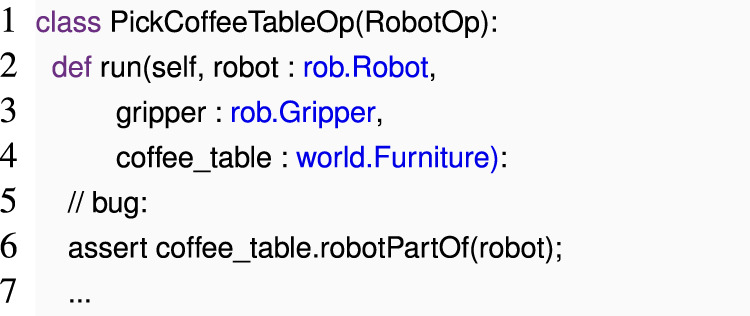


This example is derived from the SkiROS2 ontologies, with minor simplifications. In the above SkiROS2 code, the developer intended to write a pre-condition that to be able to pick a coffee cup, the robot should be close to the table. Instead, the developer mistakenly wrote that a robot should be a part of the coffee table.

The ontology requires that robotPartOf is a relation between a technical Device and a Robot. However, Furniture is not a subtype of Device, so the assertion in line 6 is unsatisfiable.

We can again detect this bug through symbolic tracing. This time, we must construct symbolic variables for robot, gripper, and coffee_table that expose methods for all applicable relations, as described by their types. For instance, gripper will contain a method robotPartOf(gripper, obj) that records on each call that gripper and obj should be in a robotPartOf relation. Meanwhile, coffee_table will not have such an operation. When we execute run (), we can then defer to Python’s own type analysis, which will abort execution and notify us that coffee_table lacks the requisite method.

Key to this symbolic tracing is our use of mock objects as symbolic variables. Symbolic variables reify Python variables to objects that can trace the operations that they interact with, in execution order, and translate them into constraints.

The main *limitation* of this technique stems from its interaction with Python’s Boolean values and control flow, e.g., conditionals and loops. Python does not allow the Boolean operators to return symbolic values but instead forces them (at the language level) to be bool values; similarly, conditionals and loops rely on access to Boolean outcomes. Thus, when we execute the code in the form if x: …, we must decide right there and then if we should collapse the symbolic variable that x is bound to True or False. Although we can re-run the critical code multiple times with different decisions per branch, the number of runs will in general be exponential over the number of times that a symbolic variable collapses to bool.

#### 3.2.4 Source provenance tracking

The intent in early error detection in (embedded) DSLs is generally to prevent undesirable behavior. When this undesirable behavior is due to a problematic user specification, it is—in our experience—valuable to point the user to the problematic specification. In practice, “blaming” the right part of the program can be non-trivial since the disagreement may be across multiple user specifications (Ahmed et al. (2011) discussed this challenge in more detail).

Handling multiple conflicting constraints can be particularly challenging for embedded DSLs. Let us say that we are using a technique like *symbolic tracing* in two user-defined functions, namely, declaration () and implementation (), such that implementation () must *ensure* the constraints that are *required* declaration ().


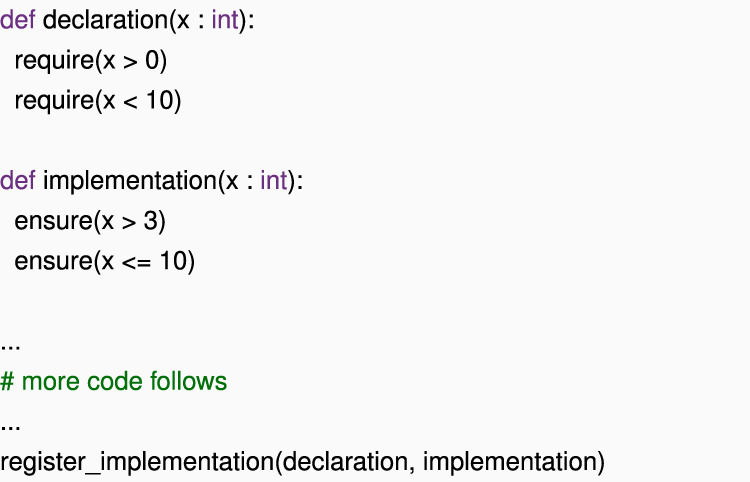


In the above example, we might find a bug: implementation allows x = 10, but this is not allowed according to declaration (). A typical but naïve implementation of such a consistency check might simply inform the user that declaration and implementation disagree about what x is allowed to do and raise an exception.

The programmer must now identify the line of code that is the culprit by hand. In practical scenarios, such as our case studies, there may be multiple declaration and implementation functions in the same file (usually as methods), which further complicates the task.

Reflection can help us here; for example, given a function object in Python, we can use reflection to access implementation.__code__.co_firstlineno and implementation.__code __.co_filename to obtain the location at which the function was defined in the form of the first line of the code and the source file name. For larger definitions, even this information may be insufficiently precise.

Some languages offer facilities that allow us to obtain even the exact lines of code that were responsible for the error (lines 3 and 7, in our example). Although some languages support this inspection through macro- or pre-processor facilities (e.g., __LINE__ and __FILE__ in C), Python 3.1 and later versions offer direct read access to the call stack via inspect. stack (). The symbolic tracing code for require () and ensure () can then “walk” this stack down until it finds the first stack frame that belongs to the code under analysis and extract file name and line number from there. The symbolic tracer can then attach this *provenance* information to the constraint and expose it to the user if the constraint is contributing to some error report.

### 3.3 Alternative techniques for checking

Internal DSLs are not the only way to implement the kind of early checking that we describe. The *mypy* tool^8^ is a stand-alone program for the type-checking Python code. *mypy* supports plugins that can describe custom typing rules, which we could use, e.g., to check for ontology types. Similarly, we could use the Python ast module to implement our own analysis over the Python source code. However, both approaches require separate passes and would first have to be integrated into the ROS launch process. Moreover, they are effectively static, in that they cannot communicate with the program under analysis; thus, we cannot guarantee that the checker tool will see the same configuration (e.g., ontology and world model).

Another alternative would be to implement static analysis over the bytecode returned by the Python disassembler dis, which can operate on the running program. However, this API is not stable across Python revisions^9^.

An external DSL such as MAESTRO Coste-Maniere and Turro (1997) would similarly require a separate analysis pass. However, it would be able to offer arbitrary, domain-specific syntax and avoid any trade-offs induced by the embedding in Python (e.g., Boolean coercions). The main downside of this technique is that it requires a completely separate DSL implementation, including maintenance and integration.

## 4 SkiROS2: an open-source software for skill-based robot execution

As a case study, we implement our patterns on the open-source software for skill-based robot execution SkiROS2 (Mayr et al., 2023b). SkiROS2 is used by several research institutions in the context of industrial robot tasks, as demonstrated in Mayr et al. (2022a), Mayr et al. (2022b), Mayr et al. (2023c), Mayr et al. (2023a), Ahmad et al. (2023), and Wuthier et al. (2021). It is a re-implementation of the predecessor SkiROS1 by Rovida et al. (2017a) and is implemented in Python on top of the robot operating system (Quigley et al., 2009) middleware. SkiROS2 uses behavior tree (Colledanchise and Ögren, 2018) formalism to represent procedures.

**FIGURE 2:**
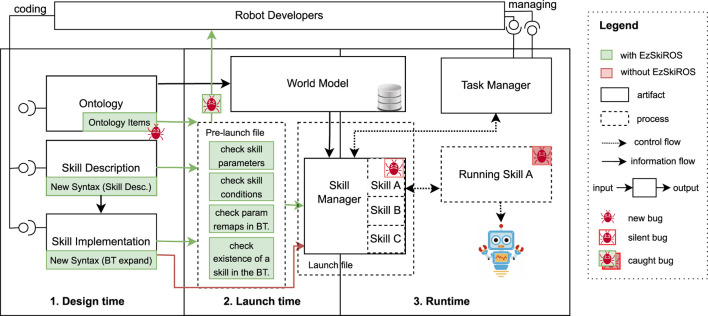
Diagram with the different components of SkiROS2, their interaction during different time phases, and the advancements by EzSkiROS (shown as green blocks). In SkiROS2, a bug that has been introduced in a skill description by a developer will often only trigger at runtime. EzSkiROS addresses these costs and risks by adding checks to find a wide range of bugs by running a pre-launch file where the skills are loaded before runtime.

SkiROS2 implements a layered, hybrid control architecture to define and execute parametric *skills* for robots (Bøgh et al., 2012), Krueger et al. (2016). The SkiROS2 system architecture is shown in Figure 2, which illustrates how different components interact with each other in various phases. It uses *ontologies* to represent the comprehensive knowledge about the world. SkiROS2 represents knowledge about the skills, the robot, and the environment in a *world model* (WM) with the *ontologies* specified in the OWL format. This explicit representation, built on the World Wide Web Consortium’s Resource Description Framework (RDF) (Hitzler et al., 2009) standard, allows the use of existing *ontologies*. This approach to knowledge management is important for complex decision-making and reasoning in autonomous systems (Cangelosi and Asada, 2022). WM is central to SkiROS2’s architecture and serves as a dynamic repository of the robot’s environment and state. It continuously updates and maintains a semantic representation of the surroundings, objects, and the robot’s own status. The integration of the WM with the *ontologies* shown in Figure 2 ensures that the robot has a thorough understanding of its operational context, enhancing its interaction capabilities with the environment.

### 4.1 Skill model

*Skills* in SkiROS2 are parametric procedures that modify the world state from an initial state to a final state according to pre- and post-conditions (Pedersen et al., 2016). Every skill has a *Skill Description* and one or more *Skill Implementation* as shown in Figure 2. The *Skill Description* consists of the following four elements:

1. *Parameters* define the input and output of a skill. The types of these parameters can vary from certain primitive data types to a *world model* element in the *ontologies.*
2. *Pre-conditions* must hold before the skill is executed.
3. *Hold-conditions* must be fulfilled during the execution.
4. *Post-conditions* indicate that a skill has been successfully executed.

These conditions are checked by the *Skill Manager* as shown in Figure 2. These conditions are important for planning and also for dynamic sanity checks, when planning is disabled. When a skill is invoked, the system first checks the pre-conditions to decide if it is safe or viable to start the skill. During execution, hold conditions are continuously monitored to ensure ongoing criteria are met. Finally, once the skill reports its completion, post-conditions are checked to confirm successful execution. These checks are essential to maintain the robustness, safety, and reliability of robotic operations, ensuring that skills are only performed when appropriate and achieve the intended results.

#### 4.1.1 Skill description

Listing 4 shows how developers define a “pick” skill in SkiROS2 by calling the Python method addParam to set the parameters of the skill and similarly to define its pre- and post-conditions. The parameters are typed, using basic datatypes (e.g., str) or a WM element defined in *ontology*, and can be *required*, *optional*, or *inferred* from the world model. Pre-conditions allow SkiROS2 to check requirements for skill execution and to automatically infer skill parameters from the world model. For example, in the “pick” skill shown in Listing 4, the parameter “Object” in line 10 is Required, i.e., it must be set before the execution of the skill. At execution time, SkiROS2 infers the parameter “ObjectLocation” (line 9) by reasoning about the pre-condition rule “ObjectLocationContainObject” (line 13). If “Object” is semantically not at a location in the WM, the pre-conditions are not satisfiable and the skill cannot be executed.

#### 4.1.2 Skill Implementation

The *Skill Implementation*, on the other hand, acts as a class that implements the interface *Skill Description* and refers to the actual coding and logic that enables a robot to perform a task. Skills can be either primitive or compound skills. Depending on the type of skill, primitive skills implement atomic functions that change the real world, such as moving a robot arm, whereas compound skills build complex behaviors in a BT. An example of a pick *Skill Implementation* is shown in Listing 5.

Listing 4 An excerpt of the parameters and pre- and post-conditions of a pick skill in SkiROS2 without EzSkiROS. It depends heavily on the usage of strings to refer to parameters or classes in the ontology.

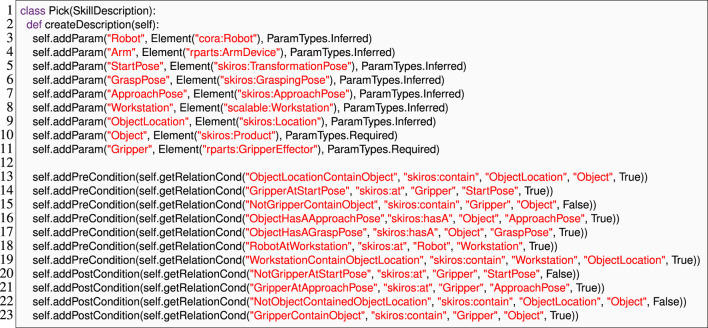

**FIGURE 3:**
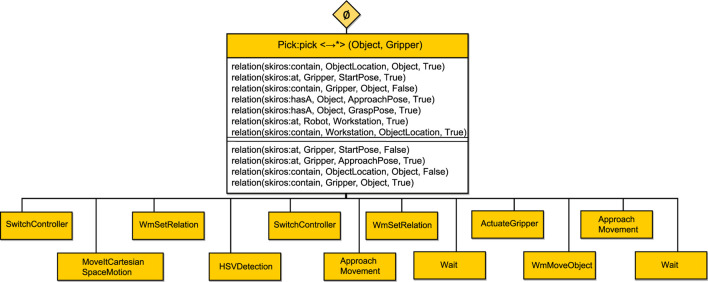
BT of the pick skill in the *eBT* format Rovida et al. (2017b). It has a *SerialStar* operator and will execute all children in sequence. The pre-conditions and post-conditions are shown.

The *createDescription* method (line 2 in Listing 5) sets the description (interface) to an implementation. The *expand* method (line 5 in Listing 5) within the skill implementation uses behavior trees to structure the execution of skills. Each node in the tree could represent a specific skill (action node) or a decision-making process (commonly known as a control flow node) that determines which skill to execute next, as illustrated in Figure 3. The control flow node sets the processor and specifies how the compound skill is decomposed into a behavior tree (line 6). In SkiROS2, control flow nodes or processors dictate how a compound skill invokes its child skills. Before delving into specific processors, it is essential to understand the common states in which a node might return during execution.

• *Success* indicates that the skill or all skills (in case of compound skills) have been completed successfully.
• *Failure* indicates that the skill has failed to complete successfully or conditions for success are not met.
• *Running* indicates that the skill is still in progress and has not yet reached a conclusion of success or failure.

These states are not only exclusive to compound skills but are also applicable to leaf nodes/primitive skills. Following are the lists of processors and how they operate in these states:

• *Serial* processes the children one by one in order until all succeed. It will continuously loop through the children until one returns RUNNING or FAILURE or until all children succeed. *SerialStar* is a variation of the serial processor with error handling.
• *Selector* runs its children one after the other until one succeeds (returning SUCCESS) or all fail (returning FAILURE). If a child is in progress (RUNNING), the processor will also return RUNNING. *SelectorStar* is a variation of *Selector* analogous to *SerialStar*.
• *ParallelFf* (parallel first fail) invokes all the children at the same time. It returns SUCCESS only if all children succeed. If any child fails, it immediately returns FAILURE and halts the other children.
• *ParallelFs* (parallel first stop) also runs all the children simultaneously. However, it stops all processes and returns SUCCESS as soon as any child succeeds or FAILURE if any child fails, regardless of the others’ states.

When we say that a node “returns” something, we are referring to the result of an operation or computation performed by that node. This result dictates the next action in the behavior tree, such as whether to continue, stop, or try a different approach.

Listing 5 The skill implementation of the pick *Skill Description* is shown in Listing 4.

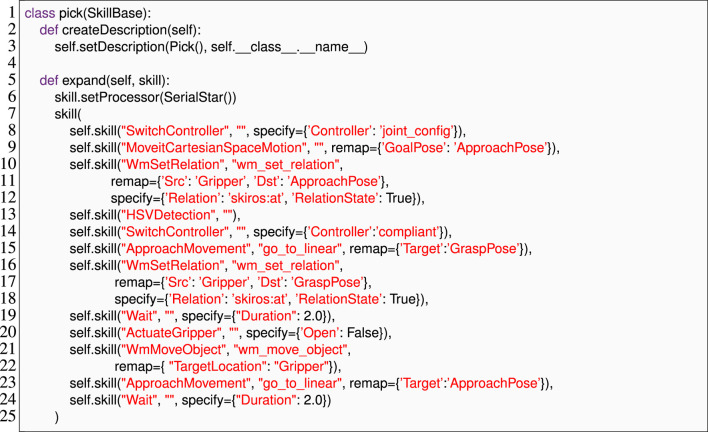

As shown in Listing 5, the skill() operator allows us to set the children of the behavior tree of the skill being implemented. To add several children at once, it is possible to use the syntax shown in the Listing 5 (lines 9–22). Each child can either be another processor (to make a nested control structure) or an individual component skill. Individual skills follow the template self.skill (skilltype, label = “ ”, specify = {}, remap = {}) self.skill (skilltype, label = “ ”, specify = {}, remap = {}), where skilltype is a *Skill Description*, i.e., an abstract skill that may have multiple implementations. At runtime, SkiROS2 selects and substitutes one of the implementations of this skill description, unless users manually select a specific implementation using the optional label parameter. All skills share a parameter namespace so that parameters with the same names are implicitly unified across all component skills. For example, if we use a compound skill with the parameter Robot set to some specific object, SkiROS2 implicitly sets this parameter in all component skills. Skill developers can override this behavior with the optional specify and remap parameters to self.skill.specify takes a Python dictionary that maps parameter names to concrete values (e.g., the Duration of a Wait action, in line 19 of Listing 5). Meanwhile, remap maps parameter names to the names of other parameters. Considering line 15 shown in Listing 5, this line specifies that the parameter Target of the ApproachMovement skill should be the parameter GraspPose, whereas the same parameter for the same skill in line 23 should be the parameter ApproachPose.

The relationship between *Skill Descriptions* and BTs is evident in how the *expand* function uses the behavior tree structure to implement the skill logic. The parameters, pre-conditions, hold-conditions, and post-conditions defined in the *Skill Description* guide the construction and execution of BTs. For instance, the pre-conditions in a skill description determine when a particular branch of the behavior tree is activated, and the post-conditions signal when a skill or sequence of skills has been successfully completed.

These skills are loaded by the *Skill Manager* at robot launch time (shown in Figure 2).

## 5 Case study I: concise and verifiable robot skill interface

We have validated our design patterns in an internal DSL EzSkiROS, which adds early dynamic checking (Section 3.2) to skill descriptions. Following a user-centered design methodology, we developed EzSkiROS by first identifying needs for early bug checking via semi-structured interviews with skilled roboticists who use SkiROS2, reviewed documentation, and manual code inspection. We found that even expert skill developers made errors in writing Skill Descriptions and that Python’s dynamic typing only identified bugs when they triggered faults during robot execution.

We designed EzSkiROS to simplify how Skill Descriptions are specified, with the intent to increase their readability, maintainability, and writability. We map ontology objects and relations into Python’s type system. Skill Descriptions can then directly include ontology information in type annotations. This approach streamlines the syntax by avoiding redundant syntactic elements and specifying type information through annotations rather than string encodings, as illustrated with the example of the pick skill in Listing 6. The listing also illustrates the EzSkiROS syntax for the example of the pick skill from Listing 4. The Skill Description shown in Listing 6 is more concise and intuitive, with type annotations providing a clear and direct way to specify the types of parameters and their ontology information.

Listing 6 The skill description of the pick skill is shown in Listing 4with EzSkiROS. We represent OWL classes in Python as identifiers in type declarations.

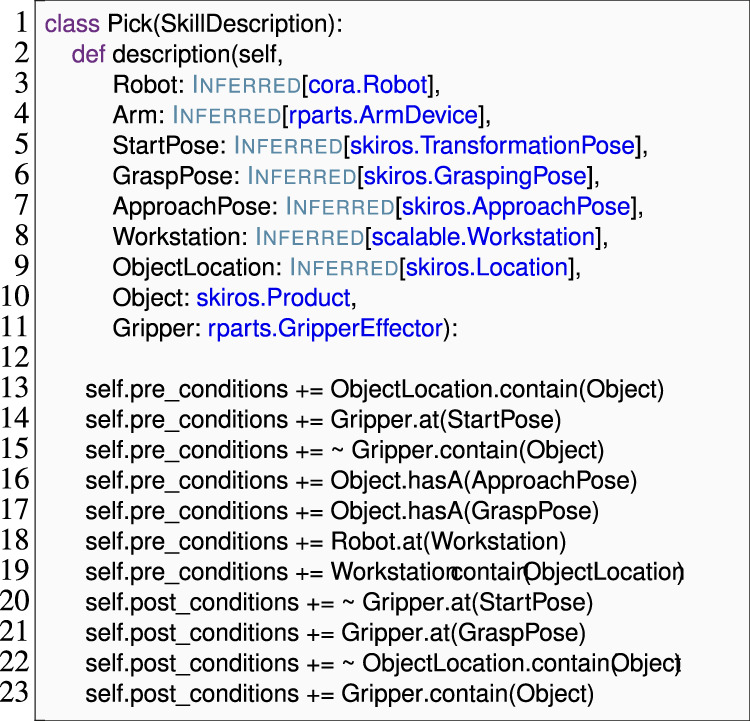

In EzSkiROS, we employed *owlready2*’s approach to domain language mapping in exposing the world model elements in the ontology as Python types and objects. For instance, as shown in Listing 6, line 3 describes a parameter Robot with the type annotation Inferred [cora.Robot]. Here, cora.Robot is a Python class that we dynamically generate to mirror an OWL class “Robot” in the OWL namespace “cora”. Inferred is a parametric type that tags *inferred* parameters. We mark *optional* parameters analogously as Optional ; all other parameters are *required*. At robot pre-launch time, we use Python’s reflection facilities to extract and check this parameter information, both to link with SkiROS2′ skill manager and for part of our early dynamic checking. In addition to our ontology types, we also allowed basic data types (str, float, int, bool) in EzSkiROS, enforcing that each must specify a default value. Originally, SkiROS2 also allowed the parameters of data types *list* and *dict*. However, in EzSkiROS, we restricted the use of *lists* and *dicts* as it was not clear if we would need this in practice. One of the developers claimed that *dicts* are considered “hacks” in the system’s context. Although *lists* are valid for representing, e.g., joint configurations, it might be better served by a specialized joint-configuration type to encapsulate their complexities and intended use more accurately. We allowed *enums* to handle such parameters, acknowledging that enums cannot encode lists or dicts, but it can provide a more controlled and predictable set of values, enhancing the system’s integrity and reliability.

In addition to skill parameters, we also want to make sure that skill conditions satisfy the contracts in our ontology. These pre-, post-, and hold-conditions can be expressed in different ways depending on what aspects of the robot’s environment and state we want to assess. According to SkiROS2 documentation, one can define a skill with the help of four kinds of skill conditions:

1. *ConditionHasProperty* is a unary relation to check whether a certain element or entity has a specific property. It is useful when the skill needs to verify certain attributes or characteristics of objects or elements before proceeding. When a condition checks for a property, it is essentially querying the ontology to see if the entity conforms to certain criteria or states defined within it. For instance, if an ontology defines that a “door” entity can have a *state* property with values “open” or “closed,” *ConditionHasProperty* might check if the door’s state is “open.”
2. *ConditionProperty* is a binary relation which relies on the ontology to understand and evaluate properties or attributes of entities. However, it might be used to assess the value or state of a property rather than just its presence. For example, it could check whether the temperature (property) is within a certain range.
3. *ConditionRelation* is used to evaluate the relationships between different elements or entities. It is crucial for tasks that require understanding spatial or hierarchical relationships, such as “is next to,” “is on top of,” or “is part of.” This condition utilizes the relational information in the ontology to assess how entities are related. *Ontologies* define not just entities but also the possible relationships between them. For example, it might check if “object A is on top of object B” by referring to the ontology’s definitions of “object A,” “object B,” and “on top of” relations.
4. *AbstractConditionRelation* is a more generalized or template form of *ConditionRelation*, which can be specified or extended for various specific relational conditions.

Since all types of skill conditions rely heavily on the ontology for their evaluation, it is important to add **Early Dynamic checking** to detect mistyped conditions. We utilize **Symbolic Tracing** as described in Section 3.2. This step collects all pre-, post-, and hold conditions via the overloaded Python operator “+=” (lines 13–23). We then check for wrong ontology relations and ontology type errors among these conditions. Since we use **Domain Language Mapping** to expose the world model entities as classes and relations as Python methods, Python’s own name analysis will catch such mistyped ontology relation or entity names, and the symbolic values that we pass into the description method capture all types of information that we need for type-checking.

We test our DSL implementation by integrating it with SkiROS2 to see how it behaves with a real skill running on a robot^10^. To demonstrate the effectiveness of our type check in EzSkiROS, we use a “pick” skill written in EzSkiROS (Listing 6) and load it while launching a simulation of a robot shown in Figure 1.

Listing 7 shows that the *ObjectProperty* “hasA” is a relation allowed only between a “product” and a “TransformationPose”. If we introduce a nonsensical relation like Object.hasA (Gripper), then the early dynamic check in EzSkiROS over ontology types returns a type error:

TypeError: Gripper: <class ’ezskiros.param_type_system.rparts.GripperEffector’> is not a skiros.TransformationPose

In addition to the error message, we also provide the source of the error highlighting the line that contains the error.

### 5.1 Evaluation

To evaluate the effectiveness and usability of EzSkiROS in detecting bugs at pre-launch time, we conducted a user study with robotics experts. Seven robotic skill developers participated in our user study, including one member of the SkiROS2 development team. The user study consisted of three phases: an initial demonstration, a follow-up discussion, and a feedback survey^11^. Due to time limitations, we defer a detailed study, with exercises for users to write new skills in EzSkiROS, to the future.

To showcase the embedded DSL and the early bug checking capabilities of EzSkiROS, we presented a video showing 1) a contrast between the old and new skill descriptions written in EzSkiROS and 2) demonstrating how errors in the skill description are detected early at pre-launch time by intentionally introducing an error in the skill conditions.

During the follow-up discussion, we encouraged participants to ask any questions or clarify any confusion they had about the EzSkiROS demonstration video.

After the discussion, we invited the participants to complete a survey to evaluate the readability and effectiveness of the early ontology type checks implemented in EzSkiROS. The survey included Likert-scale questions about *readability*, *modifiability*, and *writability*. Six participants answered “strongly agree” that EzSkiROS improved readability, and one answered “somewhat disagree.” For modifiability, four of them “strongly agree,” but three participants answered “somewhat agree” and “neutral.” All the participants answered “strongly agree” or “somewhat agree” that EzSkiROS improved writability.

To gain more in-depth insights, the survey also included open-ended questions, e.g., a) “Would EzSkiROS have been beneficial to you, and why or why not?”; b) “What potential benefits or concerns do you see in adopting EzSkiROS in your work?;” and c) “What potential benefits or concerns do you see in beginners, such as new employees or M. Sc. students doing project work, adopting EzSkiROS?”

For question a), all participants agreed that EzSkiROS would have helped them. Participants liked the syntax of EzSkiROS, and they thought that it takes less time to read and understand the ontology relations than before. One of them claimed that “pre- and post-conditions are easy to make sense.” They also found that mapping the ontology to Python types would have helped reduce the number of lookups required in the ontology. One of the participants said, “in my experience, SkiROS2 error messages are terrible, and half the time they are not even the correct error messages (i.e. they do not point me to the correct cause), so I think the improved error reporting would have been extremely useful.”

For question b), the majority of participants reported that EzSkiROS’s concise syntax is a potential benefit, which they believe would save coding time and effort. One participant found EzSkiROS’s specific error messages useful, responding that “the extra checks allow to know some errors before the robot is started,” while one participant answered that EzSkiROS does not benefit their current work, but it might be useful for writing a new skill from scratch. None of the participants expressed any concerns about adopting EzSkiROS in their work.

Listing 7 The definition of the object property “has A” in the SkiROS2 ontology.

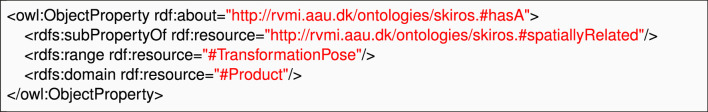

For question c), one developer acknowledges the benefits of EzSkiROS by saying “In addition to the error reporting, it seems much easier for a beginner to learn this syntax, particularly because it looks more like “standard” object oriented programming (OOP.)” One person claimed that EzSkiROS would help beginners, describing SkiROS2 as “it is quite a learning curve and needs some courage to start learning SkiROS2 from the beginning autonomously.”

In summary, the results of the user evaluation survey indicate a positive perception of EzSkiROS in terms of readability and writability. Most respondents found EzSkiROS to be easy to read and understand, with only one exception. In addition, respondents found EzSkiROS’s early error checking to be particularly useful in detecting and resolving errors in a timely manner. This suggests that the users perceived EzSkiROS as an effective tool.

## 6 Case study II: verifiable construction of a behavior tree in skill implementation

In the second case study, we examined the utility of our design patterns by extending EzSkiROS to add **Early Dynamic Checking** to the implementation of compound skills. Compound *Skill Implementation* uses behavior trees to efficiently handle decision-making processes, task execution, and error recovery. Our design methodology involved identifying the requirements for constructing BTs by examining their specifications. To understand common challenges, we analyzed GitHub issues encountered by developers when writing BTs in SkiROS2. This analysis included a systematic search for specific keywords such as “Behavior Tree,” “Remaps,” and “Skill Implementation,” informed by insights from senior Ph.D. students. Subsequently, we engaged in a verification process with the developers to ensure the validity of the identified issues.

We found that past mistakes in BT construction involved mistyped skill names and parameter names (cf. Listing 5), especially in parameter remapping. We additionally identified the concern that the pre-conditions and post-conditions of skills might be mismatched, which we explore in Section 6.1.

As shown in our previous case study, we used **Domain Language Mapping** to identify mistyped names in skill implementations early. Since the parameters to each skill implementation are defined in the skill description that is being implemented, this mapping required us to link each implementation to its corresponding description. Existing SkiROS2 code relied on calls to a setDescription () method to dynamically establish this relationship, as shown in line 2 of Listing 5. In practice, each skill implementation has exactly one skill description that it implements, meaning that there is no need to dynamically set this property. Instead, this relationship is closely related to the concepts of subtyping and interface implementation. We thus applied **Domain Language Mapping** to use Python’s syntax for inheritance as a device for specifying the link from skill implementation to skill description (as shown in Listing 8; line 1). This approach both shortened the specification and allowed us to reliably identify the parameters and conditions (pre-conditions, post-conditions, etc.,) for each skill implementation.

Recall from the discussion shown in Section 4.1.2 how behavior trees are constructed in the *Skill Implementation* phase. Behavior trees were specified in the *expand* method where a list of skills is passed to a skill() wrapper after initializing a processor (lines 7–24). Each skill is defined with self.skill (skilltype, label = “ ”, remap = .., specify = ..), allowing for parameter remapping. While composing skills in a behavior tree, the skills, their implementations, and the parameter remappings were passed as string parameters. For example, the BT specification for a “pick” skill in Listing 5 consists of a skill ApproachMovement.go_to_linear as self.skill (“ApproachMovement”, “go_to_linear”, remap = ’Target’:’Grasp-Pose’). There are two problems with this notation that could lead to a runtime error: 1) if we pass a string that does not match any available skill descriptions or its implementations, and 2) incorrect remapping, such as referencing non-existent parameters, can lead to errors. Remapping is critical as it redirects parameters from one skill to another, ensuring proper data flow.

To prevent unexpected behavior at runtime, it is vital to detect and report such errors early. To address these issues, we use **Domain Language Mapping** to expose skill descriptions, implementations, and their parameters as Python objects and passed as identifiers (as shown in line 10 of Listing 8) that allow us to use Python’s name analysis to locate skills with correct parameters (to remap to) and to find typos in those identifiers. Listing 8 shows how “pick” skill parameters are passed to the expandBT method and accessed directly as params.ApproachPose. This approach simplifies parameter remapping, ensuring accuracy and cohesiveness in skill execution.

Listing 8 The EzSkiROS representation of the skill implementation is shown in Listing 5. Here, the inheritance from Pick.SkillBaselinks the pickskill description shown in Listing 6to its implementation.

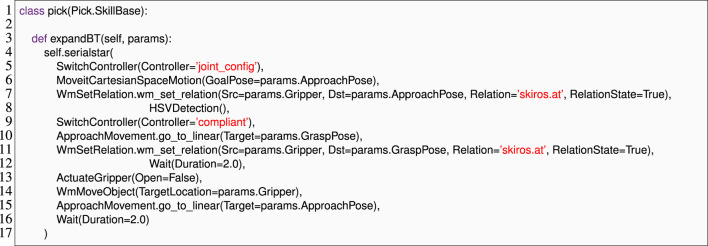

### 6.1 Need for static pre-/post-condition matching in SkiROS2

As mentioned in Sections 4, 5, pre- and post-conditions in a BT implementation of a compound skill ensure the correct execution of skills to complete a robot’s task. These conditions are checked in SkiROS2 by the *Skill Manager* before starting and parameterizing the skill. Although these conditions might seem less critical in controlled or smaller settings, their importance escalates as the complexity and scale of tasks grow. Poor quality or incorrectly defined conditions can significantly limit the ability of SkiROS2 to scale and handle complex, dynamic tasks efficiently. If we do not use a planner, manually creating compound skills or adjusting existing compounds without thorough checks can lead to mismatches between expected and actual skill behaviors. Static checking of pre-/post-conditions becomes essential to identify and correct these errors early in the development cycle, preventing potential failures during execution. To verify this requirement, we randomly selected five SkiROS2 skills written by developers to understand the prevalence of errors. Among those five skills, four of them failed the following basic checks:

• For skills in a *serial* or *serialstar* processor s = serial (A, B, C), the pre-condition of “s” must entail the pre-condition of “A,” and the aggregate post-conditions of “A” must entail the pre-condition of “B” and so on.
• For skills in a *selector* or *selectorstar* processor s = selector (A, B, C), the pre-condition of “s” must entail the conjunction of the pre-conditions of “A,” “B,” and “C.” Post-conditions of “s” can be conservatively checked as any of the children can lead to success without a predetermined order.
• For parallel skills, all children must succeed, with specific differences in handling the completion and order. This requires that no post-condition of one skill may invalidate the pre-condition of another due to the simultaneous nature of execution.

This evidence points to a common oversight in defining these conditions carefully and makes it important to have robust tooling to ensure that pre- and post-conditions are correctly matched and implemented. To address these challenges, we plan to create a comprehensive mapping and verification system in the future. This system would track all pre- and post-conditions, manage dependencies and changes, handle remapping accurately, and ensure that all conditions are consistent and verifiable at each step of the skill execution. It would be beneficial to use the design pattern **Source Provenance Tracking** to blame the exact skill whose post-condition did not match the expected state, which will make the debugging of behavior trees easier than before. It would likely involve a combination of static analysis tools, careful structuring of skill descriptions, and possibly enhancements to the SkiROS2 framework to support more robust condition checking and error reporting.

## 7 Overall evaluation of the extended EzSkiROS

Our evaluation of the extension of EzSkiROS (as mentioned in case study II) is primarily based on an in-depth review provided by an experienced SkiROS2 developer and maintainer who has used the tool for transforming the old SkiROS2 code into EzSkiROS. We requested developer feedback on various aspects of EzSkiROS, including its strengths and weaknesses, the impact on code readability and writability, the ease of code translation, the comprehensibility of errors encountered, and any general observations or suggestions they may have. The user’s experience offers valuable insights into the strengths, weaknesses, and overall impact of EzSkiROS on skill description development in robotics.

Strengths and Weaknesses: The developer highlighted several key strengths of EzSkiROS.

• Early detection of misuse: EzSkiROS enables the detection of misuse in the world model before the skills are utilized, enhancing the correctness of the code.
• Validation of naming in conditions: The tool validates naming in pre-conditions and post-conditions, ensuring consistency and correctness in element types and names.
• Improved error messaging: Compared to traditional SkiROS2, EzSkiROS provides clearer and more concise error messages.
• Readability: There is a significant improvement in the readability of skill descriptions and skill implementations of both compound and primitive skills.

However, the developer also noted a primary weakness.

• Developer productivity: Despite the aforementioned strengths, the developer expects that EzSkiROS will not provide substantial productivity benefits. The developer attributes this to the dynamic nature of most checks and the fact that world model errors abort Python execution, leading to one error being reported at a time.

Impact on Code Quality: The developer review suggests that EzSkiROS positively impacts the code quality in several ways:

• Correctness: By enforcing element types on parameters and consistent naming, the correctness of the code is improved.
• Readability and intuitiveness: The conciseness and clarity in pre- and post-conditions make the code easier to read and understand.
• Clarity in skill dependencies: The dependencies between Skill Description and SkillBase (Skill Implementation) of a skill are more apparent in the code.
• Conciseness in writing behavior trees: Writing behavior trees for compound skills have become more concise and less cluttered.

Translation Process: The developer reported that the translation of existing skill descriptions to EzSkiROS to be straightforward. The time required for translation depends on the number of skill descriptions to be converted, but it can be automated.

Error Reporting and Understanding: The user affirmed that the errors identified by EzSkiROS were sensible and contributed to a better understanding of the issues in the skill descriptions.

General Feedback: The developer acknowledged EzSkiROS as a significant step forward, particularly in moving from string-based descriptions to more natural and correct Python code. The reduction in common errors due to the validation of parameter names and world element relations was especially noted. For future work, the developer suggested the following:

• Static analysis integration: Implementing static analysis to run checks on modules and skills independently, possibly integrated with a linter, to further reduce bugs at an early stage.
• Code generation for enhanced development experience: Utilizing code generation to enable features like autocompletion and static checks during coding, particularly for the world model, to improve the development experience.

The user review provides an insightful evaluation of EzSkiROS, highlighting its strengths in improving code readability, correctness, and error messaging. The contribution of EzSkiROS to reducing common errors and improving the overall quality of skill descriptions is evident. According to the reviewer, it falls short in significantly enhancing developer productivity due to the fact that we do dynamic checks at pre-launch and the user suggests static analysis. It is important to note here that static check requires certain information (ontology and robot configuration) to be available at development time, which is not guaranteed. Modulo this caveat, we see no fundamental barrier toward using the techniques that we describe here for both pre-launch and static checks in practice, using language server or development environment plugins.

## 8 Threats to validity

### 8.1 Internal validity

EzSkiROS was evaluated on the skills implemented by Ph.D. students using SkiROS2 for research purposes. Consequently, there may be undetected errors or issues in other skills that utilize different or more extensive features of SkiROS2. Furthermore, the user study included only a small number of participants, which may not provide a comprehensive representation of all potential SkiROS2 users. This limitation could affect the reliability and generalizability of user feedback and reviews. For the initial in-depth review of EzSkiROS, only one experienced SkiROS2 developer was interviewed, and we have not yet evaluated it with more users of SkiROS2.

### 8.2 External validity

Although we expect that our design patterns can aid other Python-based robotic software, we have not validated this. Moreover, we have only validated these patterns for Python; it is an open question whether they would be effective in other languages such as Ruby or LISP.

## 9 Conclusion

In this paper, we present two analyses of different abstraction levels of robotic software and how we can use DSL design patterns to detect bugs at a pre-launch stage before runtime. Case study I demonstrated the value of our design patterns by showing how they help detect bugs in the high-level contracts between a variety of robot capabilities and the robot’s world model. Case study II expands EzSkiROS by adapting the same techniques to detecting bugs in lower-level implementation code; in our case that implementation uses a behavior tree to integrate different robot capabilities.

In exploring the relationship between the two analyses, it is important to ask the following: do they work separately, depend on each other, or are they independent yet work better together, creating a stronger combined effect than each would alone? The study shows that analysis of behavior trees (case study II) requires information about the skill parameters from the higher-level descriptions to check correct information being passed on between skills. Behavior trees also need to access the pre-, post-, and hold-conditions from the skill descriptions of the skill being implemented. On the other hand, the higher-level analysis (case study I) is stand-alone but can benefit from the BT sequencing information to suggest pre- and post-conditions to the developer. Our work demonstrates how embedded DSLs can help robotics developers detect bugs early, even when the analysis depends on data which are not available until run-time. Our evaluation with EzSkiROS further suggests that embedded DSLs can achieve this goal while simultaneously increasing code maintainability.

In our future work, we plan to collect some objective results to further substantiate our efforts. We plan to make EzSkiROS publicly available to SkiROS2 users so that people can write skills and transform their old skills into EzSkiROS, and we can get some error reports and if people find the error reports helpful. We aim to conduct an in-depth user study to explore how EzSkiROS assist users in writing skill descriptions and detecting bugs in behavior trees through pre- and post-condition matching. This study will mainly focus on understanding the user experience with EzSkiROS, particularly in terms of its usability and effectiveness in early bug detection. A significant aspect of this study will be to extend the possibility of the integration of the two analyses at different abstraction levels and see how their combination influences the bug detection process. We are particularly interested in whether this integration simplifies the process of writing error-free skill descriptions and how it impacts the overall development workflow. By analyzing the data collected from this study, we expect to gain valuable insights into the practical applications and limitations of EzSkiROS. This will not only help us in refining the tool but also contribute to the broader understanding of skill programming in robotics.

## Data Availability

The original contributions presented in the study are included in the article/Supplementary Material; further inquiries can be directed to the corresponding authors.

## Footnotes

1
https://github.com/RVMI/skiros2

2
https://corlab.github.io/dslzoo

3
https://github.com/BehaviorTree/BehaviorTree.CPP

4
https://github.com/splintered-reality/py_trees

5
https://docs.unrealengine.com/en-US/Engine/ArtificialIntelligence/BehaviorTrees

6
https://robmosys.eu/mood2be/

7 Python’s *eval* function offers similar capabilities, but as of Python 3.10, it does not seem to allow passing parameters to code objects.

8
https://mypy-lang.org/

9
https://docs.python.org/3/library/dis.html

10 Available online in https://github.com/lu-cs-sde/EzSkiROS

11 A replication of the survey https://github.com/lu-cs-sde/EzSkiROS
